# Effect of remote ischemic preconditioning on the risk of contrast-induced acute kidney injury in patients with coronary heart disease undergoing percutaneous coronary intervention

**DOI:** 10.3389/fcvm.2026.1870596

**Published:** 2026-06-12

**Authors:** Yanmin Wu, Xinxin Zhang, Peng Liu

**Affiliations:** Department of Cardiology, Affiliated Hospital of Hebei University, Baoding, Hebei Province, China

**Keywords:** contrast-induced acute kidney injury, coronary heart disease, percutaneous coronary intervention, remote ischemic preconditioning, risk

## Abstract

Contrast-induced acute kidney injury (CIAKI) is a frequent and clinically important complication after percutaneous coronary intervention (PCI). Despite standard preventive strategies including adequate hydration and the use of low- or iso-osmolar contrast media, the residual risk of CIAKI remains substantial. Remote ischemic preconditioning (RIPC), a non-invasive method involving brief cycles of ischemia and reperfusion applied to a limb, has been proposed as a potential strategy to mitigate ischemia–reperfusion injury, including in the kidneys. However, the impact of RIPC on the incidence of CIAKI after PCI in patients with CHD remains unclear. This study aim is to investigate the impact of RIPC on the incidence of CIAKI in patients with CHD undergoing PCI. A total of 484 patients who underwent elective PCI were enrolled and randomly assigned to either the RIPC or control group. The RIPC group received preconditioning twice daily for two days prior to PCI and once again two hours before the procedure. Renal function was assessed at baseline, 48 h, and one week post-PCI. The primary endpoint was the incidence of CIAKI, and the secondary endpoint was the occurrence of major adverse cardiac events (MACE) during the 90-day follow-up. Results showed that the incidence of CIAKI was significantly lower in the RIPC group compared to the control group (6.2% vs. 12.0%, *P* = 0.039), with multivariate analysis indicating RIPC as an independent protective factor for reducing CIAKI (OR = 0.338, 95% CI: 0.159–0.717, *P* = 0.005). However, there was no significant difference in the incidence of MACE between the two groups during the follow-up period (Log-rank *χ*² = 1.665, *P* = 0.126).In conclusion, RIPC appears to reduce the risk of CIAKI in patients with CHD after PCI, offering a simple, cost-effective, and non-pharmacological preventive strategy.

## 1 Introduction

Contrast-induced acute kidney injury (CIAKI), also known as contrast-induced nephropathy (CIN), is a common complication following percutaneous coronary intervention (PCI). It refers to a clinical syndrome characterized by renal function impairment after the administration of iodinated contrast media. It is typically defined as an increase in serum creatinine (Scr) of more than 26.5 µmol/L (0.3 mg/dL) within 48 h after contrast exposure, or an increase of more than 50% from baseline within one week, in the absence of other causes affecting renal function. CIAKI has become a common cause of hospital-acquired acute kidney injury. Studies have shown that CIAKI not only prolongs hospital stay and increases healthcare costs, but is also closely associated with adverse short- and long-term clinical outcomes, including increased incidence of cardiovascular events, progression of chronic kidney disease, and higher mortality, thereby significantly affecting patient prognosis (1, 2). Therefore, effective prevention of CIAKI has become an important issue in interventional cardiology.

Currently, preventive strategies for CIAKI mainly focus on perioperative hydration during PCI, reducing contrast volume, selecting low- or iso-osmolar contrast agents, and avoiding nephrotoxic drugs (1). However, the renoprotective effects of these measures remain limited in clinical practice, especially among high-risk populations, where the incidence of CIAKI remains high. In addition, although some pharmacological interventions (such as N-acetylcysteine and statins) have shown certain benefits in some studies, their effectiveness in preventing CIAKI remains controversial (3). Therefore, exploring new, safe, and effective non-pharmacological interventions is of great clinical significance.

Current evidence suggests that contrast-induced ischemia and hypoxia of the renal medulla are key mechanisms in the development of CIAKI. After contrast administration, increased release of vasoconstrictors such as endothelin and inhibition of vasodilators such as nitric oxide (NO) lead to renal vasoconstriction and reduced renal blood flow, resulting in renal ischemia and hypoxia. This subsequently triggers oxidative stress with increased production of reactive oxygen species and induces the release of various inflammatory mediators from renal tissues, leading to oxidative stress and inflammatory responses. These processes cause tubular epithelial cell injury and apoptosis, ultimately resulting in CIAKI, manifested as increased Scr and decreased urine output after PCI (4, 5). Therefore, improving renal ischemia and hypoxia, suppressing inflammation, and reducing oxidative stress injury are key strategies to reduce the occurrence of CIAKI.

Remote ischemic preconditioning (RIPC) is a method in which brief, non-lethal ischemic stimuli are applied to a remote organ or tissue to confer protection against subsequent severe ischemia–reperfusion injury. Studies have shown that RIPC exerts protective effects on remote organs such as the heart, lungs, kidneys, brain, and intestines (6). In recent years, its potential protective role in preventing CIAKI has attracted considerable attention. Evidence suggests that RIPC can transmit protective signals through multiple signaling pathways, thereby reducing renal injury. Its renoprotective mechanisms may include inhibition of inflammatory responses, reduction of oxidative stress, activation of bradykinin receptors, and enhancement of nitric oxide synthase activity to promote NO production (7–9)^.^

Therefore, based on the pathophysiological characteristics of CIAKI and the mechanisms of RIPC, RIPC may effectively reduce the incidence of CIAKI and mitigate contrast-induced acute renal injury. Moreover, RIPC is simple, noninvasive, and low-cost, which gives it unique advantages for clinical application. However, there is still considerable controversy regarding the effect of RIPC on the risk of CIAKI in patients with coronary heart disease undergoing PCI. Therefore, this study aims to investigate the impact of RIPC on the risk of CIAKI after PCI in patients with coronary heart disease through a prospective randomized controlled study.

## **2** Materials and Methods

### 2.1 Study population

**Figure 1:**
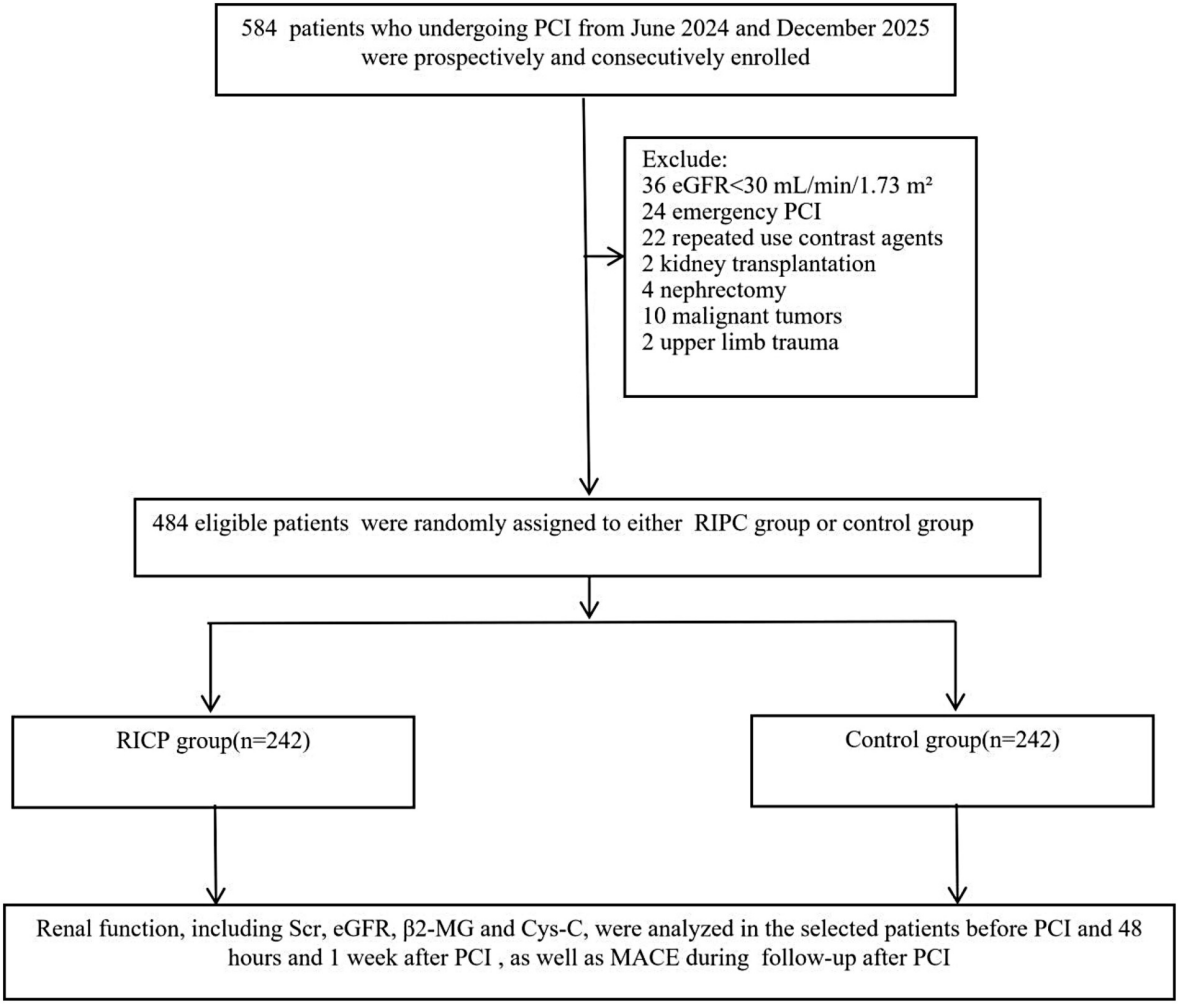
The Flowchart of this study.PCI, percutaneous coronary intervention; eGFR, estimated glomerular filtration rate; Scr,serum creatinine; Cys-C, cystatin C;*β*2-MG,*β*2-microglobulin; MACE, major adverse cardiac events; RIPC, remote ischemic preconditioning.

Patients with coronary heart disease who were scheduled to undergo elective PCI in the Department of Cardiology at the Affiliated Hospital of Hebei University between June 2024 and December 2025 were prospectively and consecutively enrolled. Inclusion criteria: (1) Meeting the diagnostic criteria for coronary heart disease; (2) Meeting the indications for PCI; (3) Age ≥ 18 years. Exclusion criteria: (1) Patients with contrast media allergy or those who had received contrast agents within one week prior to the procedure; (2) Patients with malignant tumors; (3) Patients undergoing emergency PCI; (4) Patients with cardiogenic shock, severe heart failure, or left ventricular ejection fraction (LVEF) < 30%; (5) Patients with severe renal insufficiency [estimated glomerular filtration rate (eGFR) < 30 mL/(min·1.73 m²)] or those receiving dialysis; (6) Patients with upper limb trauma or arteriovenous thrombosis; (7) Patients with a history of nephrectomy or renal transplantation; (8) Patients with psychiatric disorders who were unable to cooperate with the study or who refused follow-up. A flowchart of the study process is shown in Figure 1.

### 2.2 Study protocol

According to the order of enrollment, patients were assigned to the RIPC group or the control group based on numbers generated by simple randomization (odd numbers for the RIPC group and even numbers for the control group). A designated investigator was responsible for randomization and record keeping, while both the participants and the investigators involved in the study were blinded to the group assignments. RIPC group: Patients received remote ischemic preconditioning twice daily for two consecutive days before PCI, and once again 2 h prior to PCI, for a total of five sessions. The specific RIPC protocol was as follows: pressure cuffs placed on both upper arms were inflated to 200 mmHg for 5 min, followed by complete deflation for 5 min to allow reperfusion. This ischemia–reperfusion cycle was repeated 5 times. Control group: Patients received a sham intervention in which pressure cuffs on both upper arms were inflated to 30 mmHg (insufficient to induce ischemia) for 5 min, followed by complete deflation for 5 min. This cycle was also repeated 5 times, and the entire intervention from initiation to completion of the last cycle was scheduled to be completed within 1 h.

All enrolled patients received hydration therapy with normal saline 6–12 h before PCI and 6–12 h after PCI. To minimize the occurrence of CIAKI, all patients were administered either the low-osmolar contrast agent ioversol or the iso-osmolar contrast agent iodixanol. Relevant medications were prescribed at the discretion of the attending physicians according to the patients’ clinical condition after admission and in accordance with relevant clinical guidelines. Data collected for both groups included baseline characteristics, medication history, contrast volume, and details of PCI treatment. Renal function parameters were recorded before PCI and at 48 h and 1 week after PCI, including blood urea nitrogen (BUN), serum creatinine (Scr), estimated glomerular filtration rate (eGFR), cystatin C (Cys-C), and blood *β*2-microglobulin (*β*2-MG).

The primary endpoint was the incidence of CIAKI, defined as an increase in Scr of more than 26.5 µmol/L (0.3 mg/dL) above baseline within 48 h after contrast administration, or an increase in Scr of more than 50% above baseline within 1 week (1).All enrolled patients underwent CIAKI risk assessment according to the CIAKI risk scoring system proposed by Mehran R et al. (10). The secondary endpoints were changes in renal function before and after PCI, as well as the occurrence of major adverse cardiac events (MACE) during follow-up. All enrolled patients were followed up regularly in the outpatient clinic for at least 90 days. The occurrence of MACE during follow-up, including death, nonfatal myocardial infarction, nonfatal stroke, repeat revascularization due to angina, and acute heart failure, was recorded for all patients. The eGFR was calculated using the following formula: 186  ×  （Scr/88.4）（*μ*mol/L）^−1.154 ^ ×  age (years)^−0.203 ^ ×  （0.742 for females. Anemia was defined as hemoglobin <120 g/L in adult males and <110 g/L in females.

This study was conducted in accordance with the Declaration of Helsinki. Written informed consent was obtained from all enrolled patients, and all patients also signed informed consent for the PCI procedure. The study protocol was approved by the Medical Ethics Committee of the Affiliated Hospital of Hebei University (Approval No. HDFYLL-IIT-2025-036).

### 2.3 Statistical methods

SPSS 19.00 statistical software was used for data analysis. Continuous variables are expressed as mean ± standard deviation (x¯ ± s), and comparisons between groups were performed using the independent-samples t test. Categorical variables are presented as number of cases (%), and comparisons between groups were conducted using the chi-square test. Multivariate binary logistic regression analysis was performed to evaluate the effect of RIPC on the occurrence of CIAKI. The Log-rank test was used to compare differences in the incidence of MACE between the two groups. Kaplan–Meier survival curves were generated using GraphPad Prism 10.0 statistical graphing software. A two-sided *P* < 0.05 was considered statistically significant.

According to previous literature, the incidence of CIAKI was assumed to be 5% in the RIPC group and 15% in the control group. Using a two-sided test with a significance level of *α* = 0.05 and a statistical power of 1−*β* = 0.95, the sample size was calculated using the formula for comparing two independent proportions. After accounting for a 15% loss to follow-up, the required sample size was estimated to be at least 189 patients in each group, with a total minimum sample size of 378 patients.

## 3 Results

### 3.1 Comparison of basic characteristics between the Two groups

According to the inclusion and exclusion criteria, a total of 484 patients undergoing PCI were enrolled in this study, including 242 patients in the RIPC group and 242 patients in the control group. Among them, there were 300 males and 196 females, with a mean age of (69.44 ± 8.68) years. All patients completed follow-up as scheduled, with no loss to follow-up. The CIAKI risk score and NT-ProBNP levels in the RIPC group were significantly higher than those in the control group (*P* < 0.05), while there were no statistically significant differences in the other indicators (*P* > 0.05). (see Table 1).

**Table 1:** Comparisons of baseline characteristics between 2 group.

Variables	RIPC group (*n* = 242)	Control group (*n* = 242)	*t*(*χ*^2^)	*P*
Age （$\bar{x}$±s，years）	73.25 ± 10.26	74.28 ± 10.96	1.067^a^	0.286
Male［n（%）］	138（57.0）	146（60.3）	0.545	0.518
Smoking［n（%）］	96（39.7）	89（36.8）	0.429	0.575
Alcohol consumption［n（%）］	78（32.2）	80（33.1）	0.038	0.923
Body mass index（$\bar{x}$±s，kg/m2）	24.66 ± 3.48	25.02 ± 3.59	1.120^a^	0.263
Hypertension［n（%）］	124（51.2）	132（54.5）	0.531	0.524
Acute myocardial infarction［n（%）］	84（34.7）	78（32.2）	0.334	0.630
Stroke［n（%）］	65（26.9）	71（29.3）	0.368	0.613
Anemia［n（%）］	41（16.9）	45（18.6）	0.226	0.721
LVEF（$\bar{x}$±s，%）	51.28 ± 11.14	52.44 ± 12.36	1.084	0.279
LVEF < 45%［n（%）］	98（40.5）	89（36.8）	0.706	0.455
Single-vessel target lesion［n（%）］	69（28.5）	72（29.8）	0.090	0.841
Multi-vessel (≥2 vessels) target lesions［n（%）］	173（71.5）	170（70.2）	0.090	0.841
Number of stents（$\bar{x}$±s）	3.22 ± 1.78	3.46 ± 1.52	1.595	0.111
Ioversol ［n（%）］	108（44.6）	116（47.9）	0.532	0.466
Iodixanol［n（%）］	134（55.4）	126（52.1）	0.532	0.466
Contrast volume （$\bar{x}$±s，mL）	176.52 ± 68.43	174.39 ± 65.52	0.350	0.727
Hydration volume（$\bar{x}$±s，mL）	1,274.52 ± 365.42	1,265.55 ± 361.96	0.271	0.786
CIAKI risk score（$\bar{x}$±s，points）	12.18 ± 5.02	11.04 ± 4.62	2.599	0.010
NT-ProBNP（$\bar{x}$±s，pg/mL）	665.42 ± 217.36	615.36 ± 204.72	2.608	0.009
Fasting blood glucose（$\bar{x}$±s，mmol/L）	7.04 ± 1.88	6.76 ± 1.56	1.783	0.075
Hemoglobin（$\bar{x}$±s，g/L）	115.30 ± 30.32	117.61 ± 30.79	0.832	0.406
Triglycerides（$\bar{x}$±s，mmol/L）	1.96 ± 0.88	1.91 ± 0.82	0.647	0.518
Total cholesterol（$\bar{x}$±s，mmol/L）	5.64 ± 1.82	5.54 ± 1.75	0.616	0.538
High-density lipoprotein（$\bar{x}$±s，mmol/L）	1.72 ± 0.64	1.68 ± 0.59	0.715	0.475
Low-density lipoprotein（$\bar{x}$±s，mmol/L）	3.49 ± 1.58	3.47 ± 1.42	0.146	0.884
Statins ［n（%）］	208（86.0）	216（89.3）	1.218	0.334
*β*-blockers［n（%）］	144（59.5）	136（56.2）	0.542	0.519
ACEI/ARB［n（%）］	123（50.8）	118（48.8）	0.207	0.716
Diuretics［n（%）］	99（40.9）	80（33.1）	3.200	0.090
Calcium channel blockers ［n（%）］	117（48.3）	126（52.1）	0.669	0.467

RIPC, remote ischemic preconditioning; LVEF, left ventricular ejection fraction; CIAKI, contrast-induced acute kidney injury; eGFR, estimated glomerular filtration rate; ACEI/ARB, angiotensin-converting enzyme inhibitors/angiotensin II receptor blockers.

### 3.2 Changes in renal function before and after PCI at 48 hours and 1 week

There were no statistically significant differences between the two groups in pre-PCI levels of Scr, eGFR, *β*2-MG, and Cys-C (*P* > 0.05).At 48 h after PCI, there were no significant differences in Scr and eGFR between the RIPC group and the control group; however, the levels of *β*2-MG and Cys-C were lower in the RIPC group than in the control group, and the differences were statistically significant (*P* < 0.05).At 1 week after PCI, there were no statistically significant differences between the two groups in Scr, eGFR, *β*2-MG, and Cys-C levels (*P* > 0.05). (see Table 2).

**Table 2:** Changes in renal function before and after PCI at 48 hours and 1 week in both groups.

Variables	RIPC group (*n* = 242)	Control group (*n* = 242)	*t*	*P*
Scr（$\bar{x}$±s，μmol/L）				
PCI Pre-treatment	87.14 ± 38.04	88.78 ± 39.42	0.466	0.642
PCI 48 Hours Post-treatment	90.54 ± 40.79	91.82 ± 40.88	0.345	0.730
PCI 1 Week Post-treatment	89.08 ± 39.88	90.25 ± 39.25	0.325	0.745
eGFR（$\bar{x}$±s，mL/min）				
PCI Pre-treatment	89.26 ± 17.32	89.54 ± 17.16	0.179	0.858
PCI 48 Hours Post-treatment	86.64 ± 16.85	85.55 ± 15.44	0.742	0.458
PCI 1 Week Post-treatment	87.15 ± 16.36	86.88 ± 16.14	0.183	0.855
Cys-C（$\bar{x}$±s，mg/L）				
PCI Pre-treatment	2.25 ± 1.02	2.14 ± 0.98	1.210	0.277
PCI 48 Hours Post-treatment	3.05 ± 1.24	3.38 ± 1.32	2.835	0.005
PCI 1 Week Post-treatment	2.72 ± 1.15	2.84 ± 1.24	1.104	0.270
β-2MG（$\bar{x}$±s，mg/L）				
PCI Pre-treatment	2.36 ± 0.94	2.26 ± 0.87	1.215	0.225
PCI 48 Hours Post-treatment	3.05 ± 1.26	3.35 ± 1.37	0.507	0.012
PCI 1 Week Post-treatment	2.84 ± 1.08	2.95 ± 1.14	1.090	0.276

Scr, serum creatinine; eGFR, estimated glomerular filtration rate; Cys-C, cystatin C; β-2MG, β2-microglobulin; PCI, percutaneous coronary intervention.

### 3.3 Incidence of CIAKI

Among the two groups, a total of 44 cases of CIAKI occurred, with an overall incidence of 9.1%. In the RIPC group, 15 cases of CIAKI were observed, with an incidence of 6.2%, whereas in the control group, 29 cases occurred, with an incidence of 12.0%. The difference in the incidence of CIAKI between the two groups was statistically significant (*χ*² = 4.900, *P* = 0.039).

### 3.4 Multivariate binary logistic regression analysis

Using potential factors influencing the occurrence of CIAKI as independent variables and the presence or absence of CIAKI as the dependent variable, a multivariate binary logistic regression analysis was performed. The results showed that RIPC was an independent protective factor for CIAKI (OR = 0.338, 95% CI: 0.159–0.717, *P* = 0.005), as shown in Table 3.

**Table 3:** Multivariate logistic regression analysis of influencing factors for CIAKI.

Variables	*B*	*SE*	*Waldχ^2^*	*P*	*OR*	95%*CI*
Age	−0.029	0.035	0.682	0.409	0.972	0.908～1.040
CIAKI risk score	0.193	0.057	11.413	0.001	1.213	1.085～1.358
NT-proBNP	1.371	0.500	7.527	0.006	3.940	1.479～10.494
Contrast volume	0.666	0.389	2.935	0.087	1.947	0.908～4.172
eGFR	0.028	0.129	0.048	0.827	1.029	0.799～1.324
Iodixanol	0.699	0.690	1.028	0.311	2.012	0.521～7.777
RIPC	−1.085	0.384	7.998	0.005	0.338	0.159～0.717

RIPC, remote ischemic preconditioning; CIAKI, contrast-induced acute kidney injury; eGFR, estimated glomerular filtration rate.

### 3.5 Incidence of MACE

During the 90-day follow-up period from study initiation to the final follow-up, the occurrence of MACE was recorded in both groups. In the RIPC group, there were 2 cases of recurrent acute myocardial infarction, 5 cases of repeat revascularization, 4 cases of acute heart failure, and 2 cases of stroke. In the control group, there were 3 cases of recurrent acute myocardial infarction, 8 cases of repeat revascularization, 6 cases of acute heart failure, and 3 cases of stroke.

**Figure 2:**
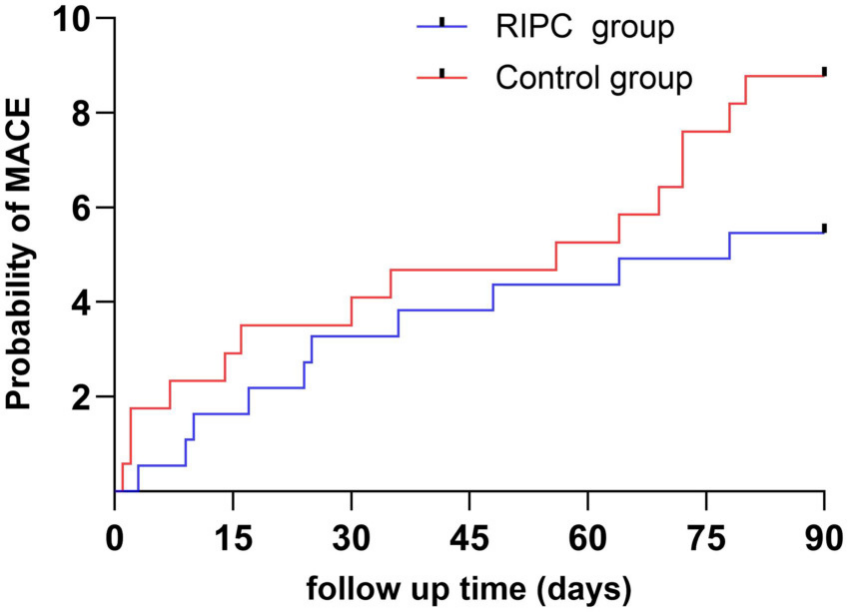
The Kaplan–meier survival curves for the RIPC group and the control group. MACE, major adverse cardiovascular events; RIPC, Remote ischemic preconditioning.

Kaplan–Meier curves were constructed to assess the cumulative incidence of MACE in the two groups, and the Log-rank test was performed. There was no statistically significant difference in the incidence of MACE between the RIPC group and the control group (Log-rank *χ*² = 1.665, *P* = 0.126), as shown in Figure 2.

## 4 Discussion

In this prospective randomized study, we found that RIPC significantly reduced the incidence of CI-AKI in patients with CHD undergoing PCI. In addition to a lower rate of CI-AKI, patients in the RIPC group exhibited smaller post-procedural increases in *β*2-MG and Cys-C, suggesting a consistent renoprotective effect. These findings support the hypothesis that RIPC can serve as an effective adjunctive strategy for renal protection in the setting of contrast exposure.

In current clinical practice, the diagnosis of CIAKI mainly relies on changes in Scr. However, Scr is influenced by multiple factors such as age, body mass index, sex, and diet, and lacks sensitivity and specificity for the early diagnosis of CIAKI. Cys-C, also known as cysteine protease inhibitor C, is less affected by muscle mass, age, and sex. It is freely filtered by the glomerulus, reabsorbed in the proximal tubules, and completely metabolized after reabsorption without being affected by external factors. Therefore, it is an ideal endogenous marker reflecting changes in glomerular filtration rate, especially in the early diagnosis of CIAKI, where its changes are more rapid and pronounced than those of Scr (11).Elevated serum *β*2-MG levels often indicate impaired glomerular filtration function. When the glomerular filtration rate decreases, *β*2-MG cannot be effectively filtered and excreted, leading to increased blood concentrations. The degree of elevation correlates with the severity of glomerular dysfunction, and it can reflect abnormalities in glomerular filtration earlier than Scr (12). Therefore, *β*2-MG and Cys-C can serve as early biomarkers of CIAKI.

In 1986, Murry et al. (13), in a canine model, demonstrated that after 5 min of occlusion of the left circumflex coronary artery followed by 5 min of reperfusion, subsequent prolonged ischemia for 40 min resulted in a reduced myocardial infarct size. Ischemic preconditioning improved myocardial energy metabolism and attenuated myocardial injury. This led to the first proposal of the concept of ischemic preconditioning. RIPC refers to a protective strategy in which brief, non-lethal ischemia–reperfusion episodes are applied to a specific organ or tissue to induce endogenous protective mechanisms, thereby increasing the tolerance of remote organs or tissues to subsequent prolonged ischemia–reperfusion injury. The heart, brain, and kidneys have distinct physiological and functional characteristics and are particularly susceptible to ischemia, hypoxia, and reperfusion injury. In recent years, RIPC has been shown to exert potential organ-protective effects in various clinical settings, including the heart, brain, and kidneys. In the cardiovascular field, RIPC has been used to attenuate myocardial ischemia–reperfusion injury (7); in neurological disorders, it is believed to improve tolerance to cerebral ischemia and hypoxia (8); and in renal protection, RIPC is thought to help prevent acute kidney injury through multiple mechanisms, including anti-inflammatory, antioxidant, and neurohumoral regulation (9).

However, the effects of RIPC on CIAKI remain controversial across different studies (14). Er F et al. (15), in the “Renal Protection Trial,” first demonstrated that in patients with chronic kidney disease at high risk of CIAKI, intermittent upper-arm ischemia applied before PCI significantly reduced the incidence of CIAKI (40% in the control group vs. 12% in the RIPC group). Valappil et al. (16) also observed a lower incidence of CIAKI in the RIPC group among high-risk patients (contrast volume >100 mL) (22% vs. 36%). RIPC significantly reduced serum creatinine levels at 24 h, 48 h, 2 weeks, and 6 weeks after PCI, and improved eGFR. Igarashi G et al. (17) found that RIPC reduced the incidence of CIAKI in low- to moderate-risk patients, possibly through the reduction of oxidative stress. However, Stokfisz K et al. (18)reported that RIPC prior to elective PCI did not significantly reduce the incidence of CIAKI, with 2 cases (4%) in the RIPC group and 3 cases (5.9%) in the control group, showing no statistically significant difference (*P* = 0.98). Belabbas et al. (19)found that acute RIPC did not provide renal protection after contrast administration, and Roubille et al. (20) also observed no effect of RIPC on the incidence of AKI or on changes in serum creatinine and eGFR in patients with chronic kidney disease. A randomized double-blind controlled study by Balbir Singh G (21) showed that RIPC did not effectively reduce the incidence of CIAKI in patients with diabetic nephropathy undergoing elective PCI. These inconsistent findings may be attributed to several limitations, such as small sample sizes, single-center study designs, lack of standardized RIPC protocols (often limited to acute RIPC applied 5 min to 2 h before PCI), and variability in endpoint definitions. This acute RIPC protocol may be insufficient to reduce the incidence of CIAKI. Therefore, this study adopted a regimen consisting of ischemic preconditioning twice daily for two consecutive days before PCI, with an additional ischemic preconditioning session performed 2 h before PCI, which may achieve a better preventive effect.

In this study, the incidence of CIAKI in the RIPC group was significantly lower than that in the control group (*χ*² = 4.900, *P* = 0.039). At 48 h after PCI, the levels of Cys-C and *β*2-MG in the RIPC group were significantly lower than those in the control group (*P* < 0.05).Furthermore, multivariate binary logistic regression analysis showed that RIPC was an independent protective factor for CIAKI in patients with coronary heart disease after PCI. These findings suggest that, on the basis of adequate hydration, RIPC may improve acute renal function injury after interventional treatment in patients with coronary heart disease. RIPC may reduce the incidence of CIAKI and has potential as a preventive strategy for CIAKI in patients undergoing PCI.

In addition, Yamanaka T et al. (22) found that pre-PCI RIPC intervention in patients with ST-elevation myocardial infarction significantly reduced the incidence of CIAKI after PCI, with a markedly lower rate in the RIPC group compared with the control group (10.0% vs. 36.0%, *P* = 0.003). RIPC was identified as a protective factor for CIAKI (OR = 0.180, 95% CI: 0.050–0.640, *P* = 0.008).

Paramasivam G et al. (23), in a study of 420 high-risk patients, reported that RIPC significantly reduced the incidence of CIAKI (8.1% vs. 15.0%, OR = 0.540, 95% CI: 0.310–0.940, *P* = 0.027), indicating that RIPC is an effective and safe method for preventing CIAKI. Sahu R et al^.^ (24) also found that in patients with chronic kidney disease undergoing PCI, RIPC significantly reduced the incidence of CIAKI (4.4% vs. 19.04%, OR = 0.198, 95% CI: 0.087–0.452, *P* = 0.004). This study indicates that remote ischemic preconditioning is a simple and well-tolerated procedure that reduces the incidence of CI-AKI in patients with CKD stages 3–4 undergoing coronary angiography.A recent study published in the European Heart Journal (25) showed that RIPC applied prior to contrast administration reduced the incidence of AKI compared with the control group (3.2% vs. 7.6%; OR = 0.40, 95% CI: 0.17–0.94, *P* = 0.03). Jia P et al. (26) also demonstrated that delayed remote ischemic preconditioning significantly reduced the incidence of acute kidney injury in high-risk patients undergoing cardiac surgery.

Currently, the most commonly used RIPC method involves inducing ischemia in one or both limbs to achieve protection of target organs. RIPC works by applying brief, non-lethal ischemic stimuli to activate endogenous protective mechanisms, thereby providing protection against subsequent severe ischemic events. The key mechanism involves the release of endogenous protective factors triggered by remote ischemic stimulation, including bradykinin and nitric oxide, which are transported via the bloodstream to target organs, activating protective signaling pathways and reducing cellular injury (7–9).Studies have shown that microRNA-21 (miR-21), an important anti-apoptotic small RNA, plays a key role in RIPC-mediated renal protection. Pan T et al. (27), in experimental animal studies, demonstrated that RIPC can upregulate miR-21 expression by activating hypoxia-inducible factor-1*α* (HIF-1*α*) in mice. miR-21 is then transported to remote organs via exosomes, exerting anti-inflammatory and anti-apoptotic effects. The expression of miR-21 increases in a time-dependent manner after RIPC and peaks at 24 h, suggesting that pre-PCI RIPC may reduce the risk of CIAKI by upregulating anti-apoptotic microRNAs. In addition, RIPC may attenuate acute renal injury by activating the phosphoinositide 3-kinase (PI3 K) pathway, inducing nitric oxide synthase expression, and promoting the opening of mitochondrial ATP-sensitive potassium channels (28). Another potential protective mechanism of RIPC involves the activation of hypoxia-inducible factors (HIFs), which are transcription factors that regulate the expression of numerous protective genes under hypoxic conditions, thereby protecting the kidneys after contrast exposure (29).Furthermore, the protective effects of RIPC against CIAKI may also involve activation of endogenous antioxidant defense systems (30), which reduce reactive oxygen species–induced oxidative stress and help preserve renal function.

In summary, RIPC may reduce the risk of CIAKI in patients with coronary heart disease after PCI and may serve as a simple, low-cost, non-pharmacological preventive strategy. RIPC has advantages including ease of application, low cost, and high safety, and therefore holds broad potential for clinical application. However, this study has several limitations: (1) This study was a single-center cohort study with a limited sample size, and the follow-up period was only three months after PCI, which was relatively short. In addition, the CIAKI risk scores and NT-proBNP levels were higher in the RIPC group than in the control group, suggesting the possible presence of selection bias during patient enrollment. These selection biases may have had a certain impact on the study results and may have affected the true effect of RIPC on cardiovascular outcomes. (2) Patients with severe renal insufficiency, severe heart failure, hypotension, and those undergoing emergency PCI were excluded; therefore, the preventive effect of RIPC in high-risk populations for CIAKI cannot be determined. (3) There is currently no standardized protocol for ischemic preconditioning, including timing and cuff pressure, which may contribute to variability in study results. (4) Inflammatory factors and oxidative stress markers before and after PCI were not assessed in this study, the specific protective mechanism by which RIPC reduces the incidence of CIAKI could not be elucidated. (5)In this study, the CIAKI risk was assessed using the CIAKI risk scoring system proposed by Mehran R et al (10). However, current individualized risk assessments and biomarker-based evaluations for PCI patients, such as the Osaka Prognostic Score (31), were not incorporated. Therefore, the effect of RIPC on the risk of CIAKI could not be comprehensively evaluated. Therefore, the precise effect of RIPC on CIAKI after PCI in patients with coronary heart disease requires further investigation in future studies.

## Data Availability

The original contributions presented in the study are included in the article/Supplementary Material, further inquiries can be directed to the corresponding author.
